# A Novel Risk Score to the Prediction of 10-year Risk for Coronary Artery Disease Among the Elderly in Beijing Based on Competing Risk Model: Erratum

**DOI:** 10.1097/01.md.0000494749.82928.d8

**Published:** 2016-08-26

In the article “A Novel Risk Score to the Prediction of 10-year Risk for Coronary Artery Disease Among the Elderly in Beijing Based on Competing Risk Model”,^1^ which appeared in Volume 95, Issue 11 of *Medicine*, Dr. Wei Wang was not originally credited in the byline. Dr. Wang contributed significantly to the study design and writing of the article. The article has since been corrected online.
